# Issues related to sanitation failure in India and future perspective

**DOI:** 10.4103/0019-5278.55131

**Published:** 2009-08

**Authors:** S. Ganesh Kumar, S. Jayarama

**Affiliations:** Department of Community Medicine, Kasturba Medical College, Mangalore, India

Dear Sir,

The October 2008 issue of the journal discussed facts concerning NFHS-3 on sanitation failure.[1] The ignored aspect of environmental sanitation in India highlighting legal regulations for its correction was discussed in the Jan 2008 issue.[2]

Millennium Development Goals (MDGs) define improved sanitation as “connection to a public sewer, connection to a public system, pour flush latrine, simple pit latrine or ventilated improved pit latrine. The excreta disposal system is considered adequate if it is private or shared (but not public), and if it hygienically separates human excreta from human contact”.[3]

The sanitation sector is in a crisis with 40% of the world's population lacking access to even basic sanitation. Behavioral change is the critical component required to improve sanitation. It is when people use a latrine, rather than when one is constructed, that the wider benefits are realized. Similarly, it applies for practicing other aspects of cleanliness like hand washing, drinking water and eating food. A systematic review estimated that the safe disposal of excreta alone can reduce diarrheal disease by 36%[4] and a separate review of hand washing with soap on diarrhea found a 45% reduction.[5] Also it was found that sanitation could prevent childhood mortality from pneumonia and malnutrition besides diarrhea.

Political will and commitment is also required urgently to tackle the crisis. Low political priority plays out in chronic under investment which leads to adverse effects of poor sanitation. Although sanitation is the single most cost effective intervention most governments, including donors, do not count what they are spending on. The allocation of central government budgetary allocation for health sector out of the total budget remains stagnant at 1.3% of Gross Domestic Product (GDP). Besides, this proportion has progressively declined from seven to 5.5% in the States. Water supply and sanitation is one of the seven sub sectors in the health sector as given by Planning Commission of India.[3] Sanitation is interlinked and interdependent on other essential health sub sectors such as medical education, training and research, public health services and control of communicable diseases and underpins all development efforts.

In a country with 35% illiterate population, effective communication of public health related issues like sanitation is a big challenge. Besides mass media, there is a need for involvement of NGOs and community based organizations and the community to tackle the crisis of sanitation failure. Decentralized management of primary health care level is strongly advocated through Panchayati Raj Institutions (PRIs) as recommended by National Health Policy 2002. The policy also recommends convergence of management of all vertical public health programs at district level and below. However, before delegation of the responsibility and devolution of financial and administrative powers to PRIs it is essential to build capacities through interactive training sessions. Simultaneously, health functionaries also need to be oriented to adjust to a new environment and play their effective roles.[6] Thus, improvement in sanitation requires newer strategies and targeted interventions with follow-up evaluation. The goals on environmental sustainability set by MDG, by 2015, seem to be unrealistic.
